# User-Configurable Timing and Navigation for UAVs

**DOI:** 10.3390/s18082468

**Published:** 2018-07-30

**Authors:** Sigurd M. Albrektsen, Tor Arne Johansen

**Affiliations:** Department of Engineering Cybernetics, Centre for Autonomous Marine Operations and Systems, Norwegian University of Science and Technology (NTNU-AMOS), O.S. Bragstads plass 2D, 7034 Trondheim, Norway; tor.arne.johansen@ntnu.no

**Keywords:** reconfigurable sensor systems, robot navigation, sensor synchronization, drones

## Abstract

As the use of unmanned aerial vehicles (UAVs) for industrial use increases, so are the demands for highly accurate navigation solutions, and with the high dynamics that UAVs offer, the accuracy of a measurement does not only depend on the value of the measurement, but also the accuracy of the associated timestamp. Sensor timing using dedicated hardware is the de-facto method to achieve optimal sensor performance, but the solutions available today have limited flexibility and requires much effort when changing sensors. This article presents requirements and suggestions for a highly accurate, reconfigurable sensor timing system that simplifies integration of sensor systems and navigation systems for UAVs. Both typical avionics sensors, like GNSS receivers and IMUs, and more complex sensors, such as cameras, are supported. To verify the design, an implementation named the SenTiBoard was created, along with a software support package and a baseline sensor-suite. With the solution presented in this paper we get a measurement resolution of 10 nanoseconds and we can transfer up to 7.6 megabytes per second. If the sensor suite includes a GNSS receiver with a pulse-per-second (PPS) reference, the sensor measurements can be related to an absolute time reference (UTC) with a clock drift of 1.9 microseconds per second RMS. An experiment was carried out, using a Mini Cruiser fixed-wing UAV, where errors in georeferencing infrared images were reduced with a factor of 4 when compared to a software synchronization method.

## 1. Introduction

**Figure 1:**
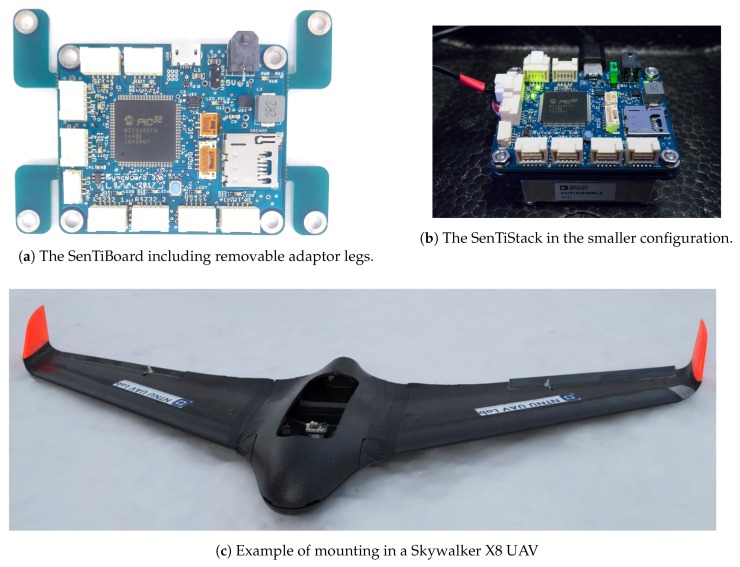
The implemented sensor timing board (SenTiBoard) in various configurations. In 1a the full-sized SenTiBoard with legs for attaching to an ODroid XU4 is shown; in 1b the SenTiStack is shown in a small configuration, without an onboard computer; and in 1c the configuration in 1b is shown mounted in a fixed wing UAV.

**Figure 2:**
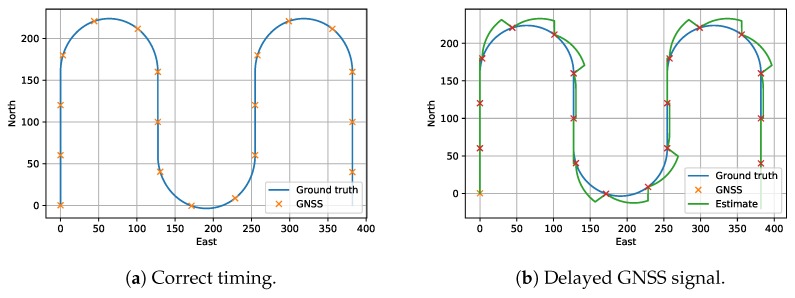
Comparison of a system with perfect timing (**a**) and a system with imperfect timing (**b**). The UAV has a velocity of 20 m s^−1^ an uncompensated GNSS time delay of 1 s. Note that this time delay is exaggerated to show the effect clearly, and is larger than an expected delay in an implemented navigation system.

Reconfigurable sensor timing hardware, that accurately records the time-of-validity (TOV) of the data, is an integral part of a highly accurate and maintainable multi-sensor navigation and image acquisition solution. TOV is often named pulse per second (PPS) in the setting of timing systems, and might be different from when it is transmitted. This especially is the case when there are large delays from the TOV to the package is transmitted from the sensor, which typically is the case for global navigation satellite system (GNSS) receivers, which need to analyze the incoming data and calculate a solution before transmitting it [1]. As many sensors, such as inertial measurement units (IMUs), measure derivatives of the desired values (acceleration, angular rate) and not the desired values (position, velocity, attitude) directly, an accurate measure of the measurement’s TOV is essential when integrating with positioning reference and image capture systems operating at high frame rates. Moreover, the high speed and fast attitude dynamics of UAVs means that time synchronization errors may have a significant impact on the measurement quality, as the measurement quality does not only depend on the value, but also the temporal accuracy of the measurement’s timestamp. Thus, hardware synchronization is required to provide the highest quality measurements possible. An implementation of such a system is depictured in Figure 1. A visualization of errors due to a time delay can be seen in Figure 2, where a simulated system with perfect inertial navigation system (INS) and GNSS sensors are integrated with and without a time delay. A very simple sensor fusion algorithm is implemented, where the position estimate is set to the GNSS value when it is received.

Developing embedded systems, especially systems without an OS, is different from writing software for modern desktop computers. Limitations on processing power, threading/interrupt behavior, real-time considerations, memory capacity, memory handling, and high requirements for stability differentiates an embedded system’s firmware from conventional software running on a PC. This makes writing correct firmware difficult. Even when considering life-critical embedded systems such as pacemakers and implantable cardioverter defibrillators, recalls or defect warnings over a one-year period is about one to 15 and nearly one to six, respectively [2], and such systems are expected to be designed to be safe and extensively tested.

### 1.1. Related Work

In the current academic state of the art, several hardware timing solutions have been used, but few have been properly described. Several publications are using some named product with little description or references to details about the hardware and methods used. Examples of such devices are the SyncBox [3], the ADAS [4,5] with the RefBox [6], the integration platform [7] and the *VIASAT* [8]. Solutions where the authors measure timestamps in a single integrated system, with specific sensors, also exist. An example is [9], where a Gumstix Overo processor running Linux with a real-time extension is used to read data from an IMU and register hardware synchronization signals from two cameras. Although all these systems seem to provide sufficiently accurate results for their use, they seem highly specialized for the task and there is little focus on maintainability and integration of different sensors.

Autopilots often aim to solve similar problems with hardware synchronization of GNSS solutions with IMU data. Examples of autopilots are the closed source CloudCap Piccolo, and the open hardware systems PIXHAWK [10] and Paparazzi [11]. The main challenge with the closed source systems is that a very limited number of sensors can be used, and integration of unsupported sensors is virtually impossible as the source code is not available. As an example, the Piccolo only supports two specified IMUs—the Crista Inertial Sensor and the Navigator GPS/INS Navigation System [12]. Integration of sensors unsupported by the open hardware systems are possible, as the firmware is available, but this can be challenging and requires expertise in embedded software development and alteration of the autopilot’s firmware, which is flight safety critical and should therefore only be altered with care.

To simplify embedded hardware development, several solutions to lower the expertise needed for creating such systems have been created. Examples of such microcontroller development systems (MDSs) are the open-source Arduino electronics platform [13], the Teensy [14], and the discontinued Intel Edison project [15]. In addition, single-board computers such as the Raspberry Pi [16] the ODroid XU4 [17] have become popular due to their small form factor and easy operation. Finally, there are traditional microcontrollers such as the Microchip PIC32 MZ [18]. An overview of the hardware capabilities of a selection of these systems is presented in Table 1. These systems have different strengths and weaknesses, with the single-board computers typically having more powerful processors, but less support for protocols channels. As described later, in Section 3, the number of input capture (IC)s is critical for accurate sensor timing, and thus the two most promising approaches are the Arduino Due and developing a system using a microcontroller. The Arduino Due is, however, lacking when it comes to the number of U(S)ART and SPI ports, but smaller systems could be created with this system.

### 1.2. Contributions

This article presents a set of required and favorable features of a reconfigurable hardware sensor timing system, that simplifies system integration when time synchronizing sensor data, without compromising sensor accuracy. Suggestions for system modularization; a data frame format; and synchronization, queueing, timing and configuration methods are proposed. By using dedicated hardware features such as IC to record the TOV from sensors, the temporal error is minimized. To verify the validity of the system design, ahardware implementation, visualized in Figure 1, was created, where acquired measurements are referenced to a 100 MHz clock, which results in a temporal resolution of 10 ns. To further simplify system integration a software library with utilities and a high-accuracy navigation payload are presented. Results from lab tests and an experiment are presented and evaluated.

This article starts by describing key terms related to sensor timing, in addition to proposing required and desired features of a sensor system integration tool in Section 2. Then, in Section 3, solutions for the challenges with sensor timing are proposed, before an implementation, named the SenTiBoard, is presented in Section 4, along with supporting software and hardware. The implemented solution is verified in two different scenarios, before the article is concluded in Section 5.

## 2. Sensor System Timing Integration

**Figure 3:**
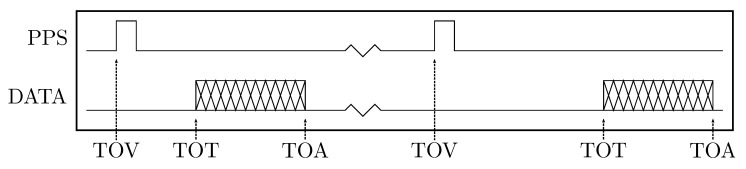
Visualization of the time-of-validity (TOV), time-of-transport (TOT) and time-of-arrival (TOA). The PPS signal line produces a rising edge when the measurement is valid, and the data is transferred after a delay. Note this delay between TOV and TOT may vary, for example due to internal calculation delays.

When associating timestamps to sensor data, there are several points in time that can be of interest. First, is the time-of-validity (TOV), which is the time at which the measurement is considered to be valid. Second, is the time-of-transport (TOT), which is when the first detectable part of the sensor message is received by the receiving platform. Finally, the time-of-arrival (TOA), which is when the full sensor message has been received, which denotes the earliest time when another algorithm can verify the sensor message’s integrity and parse and use the data. A visualization of these timestamps are given in Figure 3, where there are two transmission lines, one signalling when the data is valid, and one transferring the values measured by the sensor. In GNSS systems, the TOV is often called PPS (pulse per second) or 1PPS. We prefer the term TOV over PPS as we find it more descriptive, especially when framerates are different than 1 Hz. If we imagine an object position detection sensor, that analyses where an object is in an image, and then transfers this detection to a computer, these timestamps could be as follows. The TOV would the time be when the image was captured by the camera. Then the system would analyze the image and calculate the object’s position in the image. Then this result would be transmitted from the sensor and the TOT would be when the start of the sensor message is received at the computer. Note that this time could be significantly delayed, and that this delay could vary with many factors, such as the number of visible features in the image, the available resources on the sensor system, if other data is transferred on the same transmission line, and when the computer is ready to receive the message. The TOA would be when the whole data package has been transferred from the sensor and received at the computer. Although the TOA is the least relevant timestamp of the three, it is the one that is the easiest to implement, as one could write an application that first reads a full message from a sensor, and then assigns a timestamp to the message afterwards.

When designing a sensor timing system there are several features which are required for correct behavior: the resolution of the timestamps needs to be sufficiently high, the system must have enough interrupt capture pins to record TOVs, and the system must support the protocols used by the sensors. In addition, some sensors, such as stereo camera setups, need to triggered to achieve high-quality temporal accuracy and synchronization. When using sensors that need to stitch together several sensor readings, high-quality attitude and position estimates are essential for a satisfactory result. An example is hyperspectral cameras, which typically only reads a single line of data per frame. As even small errors in the attitude, for example in a UAV’s roll angle, will result in a large error in the measured position, especially at high altitudes, these errors must be minimized. By using sensor timing hardware, the temporal accuracy of each of the data lines can be significantly improved. By having navigation sensors connected to the same system as the hyperspectral camera, the TOV of each line from the camera can be accurately associated to the position and attitude of the UAV at the time of capture. This allows the hyperspectral lines to be transformed from the camera’s local coordinate frame, to a global coordinate frame, and simplifying the process of accurately stitching the lines together to a complete image [19].

Furthermore, the system must be able to transmit the incoming messages to a receiving platform and considerations must also be made about the format in which the sensor data is transmitted. In addition to these requirements, there are several preferred features that are not strictly required. Such features are user configurability, a minimum of 32-bit clock timer, integrated storage support, individual sensor power control, integrated sensor navigation fusion algorithms, and system status reporting.

By having a user configurable system a system integrator can for example upgrade a sensor to a newer model without requiring alterations of the hardware timing system, as long as the system supports the protocol used by the new sensor. With the rapid development in MEMS technology, new and improved sensors are released to the consumer market continuously. The effect of this can for example be seen in the Analog Device’s ADIS product line listed in Table 2, where the sensors have improved significantly over the last few years. Note that this table only considers one type of sensor typically found in a navigation payload, from a single manufacturer in a specific price range, and as sensor systems consist of several types of sensors, alterations to the system must be made almost yearly to stay up-to-date with the newest technology.

## 3. Hardware Sensor Timing

To achieve as accurate timing measurements as possible, a hardware sensor timing system is typically built around a microcontroller with specialized features for parallel registration of hardware interrupts, called input capture (IC), also known as interrupt capture. The IC-pins act as regular interrupt pins, except that in addition to triggering an interrupt subroutine (ISR) when an edge is detected, which is the normal behavior of interrupt pins, the value of the clock timer when the edge was detected is recorded. This enables a microcontroller to record the time when the value of an IC pin changes, typically on the first clock cycle after the event happened, minimizing the delay of the recorded time.

Several sensors need to be connected to the sensor timing system for it to be able to relate the different measurement timestamps to each other. By using the system to read the sensors’ data in addition to the TOVs, synchronization is simplified as the system can associate the timestamps to the sensor messages directly. This is, however, not always possible. Some sensors, for example cameras, send too much data for a typical microcontroller to process. Such sensors can still be synchronized with the other sensors, either by having the timing system send a trigger pulse to the sensor, and/or by registering a TOV signal from the sensor and then associating this trigger with the sensor message at a later stage. As cameras typically have a trigger to an external flash, which is synchronized with the image capture process, this can typically be used as a TOV measurement.

The system proposed in this article is divided into several parts. First, an overview of how the different parts of the system communicates is given, followed by a description of the data envelope, which is used to both store timing information and other necessary data about the sensor message. Then, a sensor input handler is presented, which is divided into package separation and synchronization; and timestamp association. An example of how these methods work is also given. Then suggestions for transmission of the received data to an external are made, along with some considerations for implementations on a microprocessor. Finally, two methods for configuring the hardware sensor timing board are presented.

### 3.1. Communication Overview

**Figure 4:**
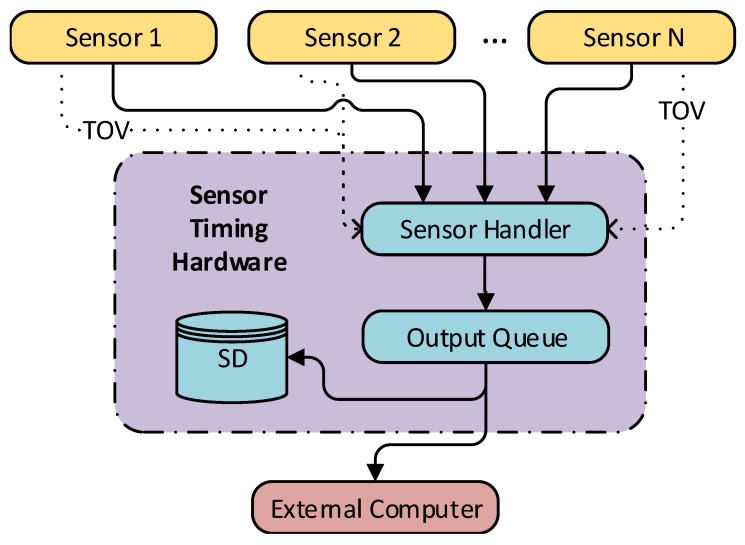
Proposed modularization of a hardware sensor timing system, which is modularized by communication.

To keep a hardware sensor timing system’s code maintainable, the authors suggest modularizing the design by creating subsystems that interact with each other only through communication channels. This design is inspired by occam’s [21] or Go’s [22] concept of channels. By modularizing the system based on how it communicates, we can modify a sub-module with minimal disruption to other sub-modules (p. 203 [23]). As the microcontroller typically runs without an operative system or a scheduler/hypervisor, it is not possible to wait for transfer channels to become ready. As the system cannot wait for communication to become ready by both the main loop and an ISR, an alternative scheme for communication must be implemented and suggestions for such systems are given later in this section. During normal operations there are two main communication channel types in the system: the transfer from a sensor to the microcontrollers internal buffer, and the transfer from the internal buffer to an external computer and/or logging to the SD-card. This system is visualized in Figure 4, with the Sensor Handler and the Output Queue modules running onboard the microcontroller.

### 3.2. Data Envelope

**Figure 5:**
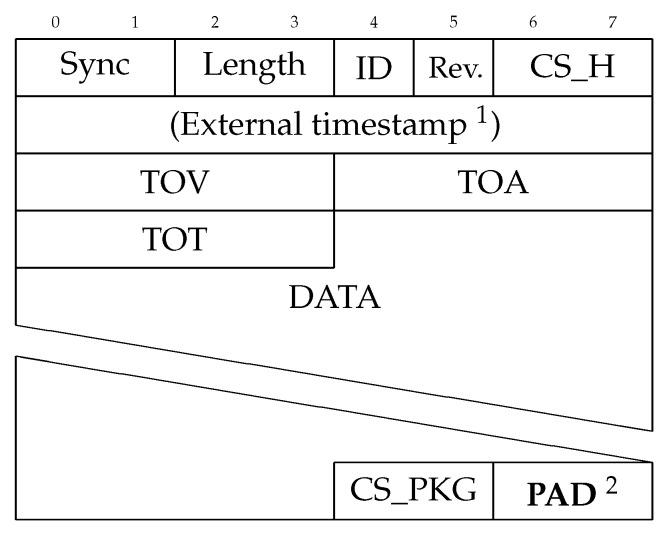
A sensor data envelope. To assign timestamps to sensor message packages, sensor data can be wrapped in this format. ${}^{1}$ An external timestamp can be added when the sensor data is transferred to an external computer. ${}^{2}$ The **PAD** bytes are zero to three extra padding bytes required by the queueing method described in Section 3.4.

To create a unified parsing system that can relate all sensor messages to a common clock, the sensor data must be associated with one or more timestamps. As not all sensors provide a timestamp as a part of the sensor message, and if a timestamp is included it is typically in the sensor’s local clock frame, a timestamp from a clock common for all the sensors must be associated with the data. To solve this, each sensor message can be wrapped in an envelope format, such as the one depicted in Figure 5. This format consists of a 8-byte header including a 2-byte header checksum (CS_H), an optional onboard timestamp (only available when logging to an onboard computer), three different timestamps, and a package checksum at the end of the package. The header is made up of a 2-byte syncword, a 2-byte unsigned integer containing the length of the package (except for the header), a 1-byte unsigned integer containing the id of the sensor port (needed to be able to distinguish multiple identical sensors), and an envelope format revision id. The header checksum’s primary objective is to ensure that the package length is correct, to prevent a very large and time-consuming read, if one bit is transferred incorrectly. After the header, the sensor message, as received from the sensor, is transmitted followed by a package checksum (CS_PKG). Both the header checksum and the package checksum are calculated using the Fletcher-8 algorithm [24]. By having a revision ID changes such as which timestamps the messages provide can be distinguished. In the presented version of the envelope there are three clock recordings: the TOV is the time-of-validity—the time when a sensor reading was validated; often referred to as PPS (Pulse-per-second) in timing applications, the TOA is the time-of-arrival—the time when a full sensor message has been transferred to the receiver, and finally the TOT is the time-of-transport—the time when the first byte of a data package is received.

### 3.3. Sensor Input Handler

There are two main methods of sensor data transfer: asynchronous transfer initiated by the sensor, which is typical for UART and UART-like communication; and polled transfer initiated by the microcontroller, which is typical for SPI communication. To create a layer of abstraction from the communication protocol, each byte received from a sensor can be passed through a sensor handler function. By designing a multi-step module that separates single sensor readings from the datastream, and registers appropriate timestamp information and associates that with each message, only a single module needs to be maintained and tested regardless of where the data was received from. Furthermore, this simplifies the configuration process, as the same configuration interface can be used for all sensor message types. A proposed sensor handler module is described below.

#### 3.3.1. Package Separation and Synchronization

To provide support for a variety of sensors simultaneously, information about how to synchronization with each sensor’s data stream needs to be configured individually for each sensor. The most common methods for synchronization of sensor data are as follows:

1. Start synchronization bytes with fixed length;
2. Start synchronization bytes with dynamic length;
3. End synchronization bytes;
4. Zero-length packages.

The first method is typically used if a sensor sends a binary stream of messages in a single format, for example from an IMU sending accelerations and angular rates. In this mode, the sensor handler module does not need to parse the length of the package from the data-stream itself as it is already specified by the format.

The second method is typically used if a sensor outputs messages of different lengths, for example a GNSS receiver that outputs satellite information. These messages typically have varying length based on how many satellites are visible at the time. To be able to use this in a binary stream, the sensor needs to provide how many bytes a package consists of—the message’s length (*L*). In this mode the sensor handler module needs information about where in a message *L* is stored, how many bytes *L* consists of and the endianness and signedness of *L*. Although a sensor message cannot have a negative length, one can imagine a format where negative values are used to indicate errors, and therefore the signness must be specified. In addition, this configuration must be able to be configured to read a specified number of additional bytes, such as header files not included in *L*.

The third method is most commonly used if the sensor is configured to send data in a human readable (ASCII) format. To avoid erroneous division of packages, the end synchronization bytes must be a series of bytes that is guaranteed to not be a part of a sensor message, except, of course, at the end. When used with a human readable format, where each line of text is a sensor reading, the newline character (\n), optionally in combination with the carriage return (\r), can be used. An example of such formats is the NMEA protocols for GNSS data  [25].

The final method is intended for use with systems that do not send any data to the sensor timing hardware itself, except for a single pulse. This is the case for camera frames that are read by an external computer, but sends a TOV message, for example from the camera’s flash output, to the sensor timing hardware. With such sensors, synchronization is trivial: as soon as a trigger is received a package with the recorded timestamp is transmitted to the sensor handler.

In addition to these methods one might imagine a fifth method using a temporal delimiter where the sensor sends a sensor measurement, followed by a pause, followed by a new distinct sensor measurement. The sensor timing system could then detect the pause, either by a timeout or by comparing a timestamp since the previous byte was received, and separate the measurement as needed. Note that the latter method is simpler to implement, but introduces a larger delay from the message has been received until it is transferred to an onboard computer.

When using the two first synchronization methods the system may become desynchronized if an error occurs in the communication link with a sensor. A desynchronized situation is detected if the bytes after reading all the package data are something else than the synchronization bytes. If this is the case, the system should raise a warning flag and skip bytes until the synchronization bytes are received. In the third and fifth modes, the system cannot be desynchronized, but the sensor can send an arbitrary long package without sending the end synchronization byte sequence. If this happens a buffer-overflow warning should be flagged and the sensor timing hardware can clear the already buffered package and start reading the next sensor message. If an operator is supervising the process, the error message can be acknowledged and cleared by him or her by sending a command to the timing system.

#### 3.3.2. Timestamp Association

Timestamp association is done in two seperate places, first after the synchronization bytes of the package has been read and then when the whole package has been transferred. The timestamp from when the first byte was received is stored as the message’s TOT, and if the sensor supports a dedicated TOV-output, the timestamp of the last received TOV for that sensor is associated with the sensor message. Note that the TOV might not update for every message. For example, if a GNSS-receiver outputs satellite observations for use with real-time kinematic (RTK)-GNSS at a rate of 10 Hz, but the navigation (and thereby time) solution is only sent at a rate of 1 Hz, the receiver might only produce a 1 Hz TOV signal (PPS). In these scenarios, the delay from TOV to TOT can be estimated using the messages with a time solution and compensated for by assuming that this delay is known and constant [1].

When the last byte of the sensor message is handled, the TOA is assigned. This value represents the earliest possible time when an application could start to process the sensor data. The TOA message is mostly used for post-process analysis of the system and is not a vital part of a real-time application.

When all the bytes of a sensor package are read, and the package has been assigned the timestamps, the information should be stored in a structured format. In this step, the total length of the data and the timestamps are copied to the correct places in the sensor data envelope (Figure 5), and checksums are calculated and inserted. By reserving space for these fields in the buffer before the sensor data is collected, additional copying due to moving the data is avoided and only the 22 bytes (8 + (3 × 4) + 2 for header, three timestamps and checksum) are written to the process. It is, nonetheless, vital that this operation is not performed in an ISR, as the checksum calculation must iterate the whole sensor package before finishing, which might take more time than is acceptable to spend in an ISR.

#### 3.3.3. Example Message

Imagine that we have a uBlox GNSS receiver connected to the sensor hardware timing board, and that it sends a short message with two bytes (in addition to its own headers) to the board. The binary sensor message could look like this: [B5 62 05 01 02 00 FF FF 1E 61], in addition let’s say that we have are not currently synchronized with the stream, and therefore we receive some other data before the message. The full message we receive is therefore: [aa aa B5 62 05 01 02 00 FF FF 1E 61 B5 62]. According to the uBlox datasheet we have configured the system to look for a synchronization id: [B5 62] and a message length in a little-endian format of two bytes, located 4 bytes into the package, with 8 bytes in addition to the length we read due to the uBlox header and the checksum. This would correspond to the second synchronization method—start synchronization with dynamic length.

Before the first part of the message is received, we register an edge on the IC-pin and the TOV is stored in a variable associated with the sensor port. Then, after some time, we receive the first byte (aa). We register the timestamp ${\mathrm{tot}}_{1}$, but as this is not the expected synchronization bytes of the uBlox protocol (B5), we do not increase the current package length, but instead flag a warning to the user. The same happens for the next byte we receive (also aa). When we receive the third byte (B5), it matches what we have configured the system for. We store the timestamp in ${\mathrm{tot}}_{1}$, and increase the current package length to 1. When we receive the next byte (62), the timestamp is stored in another value (${\mathrm{tot}}_{2}$) and as the synchronization bytes matches both in value and in length, we register that we (most likely) have achieved synchronization, increase the package length to 2 and store the value ${\mathrm{tot}}_{1}$ as TOT. We also copy the latest TOV to the sensor message. To find the length, we have to have a sensor message size at least 4 (length offset) + 2 (size of length) = 6 bytes. As we have found the TOT, we no longer need to record timestamps for the bytes.

The next few bytes—05, 01 and 02—we store in the current buffer, but when the byte after that arrives (00) we have a buffer size of 6, and we can find the expected length of the sensor message. We read the last two bytes in the buffer as a little endian integer, and we find out that we have a total expected package size of 2 + 8 = 10 bytes. We continue to read until the current package size is 10, and when we receive the last (61), we mark the sensor message as completed, we attach the TOA, and switch to another write buffer to be ready for the next sensor message message.

### 3.4. Sensor Timing Hardware Output

When a full package has been read by the sensor handler and packaged in the envelope format, the data is transferred to the data output module. This module needs to handle both small packages at a very high rate, typically IMUs with up to thousands of measurements per second, and large packages with a lower rate, for example raw data GNSS messages at ten measurements per second. Furthermore, we want the timing system to utilize direct memory access (DMA) features of the processor.

One of the most used and available protocols on the market today is the USB-protocol, which in addition to providing high transfer speeds and having the option to deliver power to attached devices, is available on almost all modern computers—both desktop computers and single-board computers such as the ODroid XU4. Through a single USB connection it is possible to have several endpoints, which may have different functions which makes it flexible. Some microcontrollers, such as the PIC32 MZ, also allow USB-transfers using DMA, which makes them a CPU-efficient protocol. Some systems, however, have limitations on which memory addresses can be accessed when using USB through DMA.

**Figure 6:**
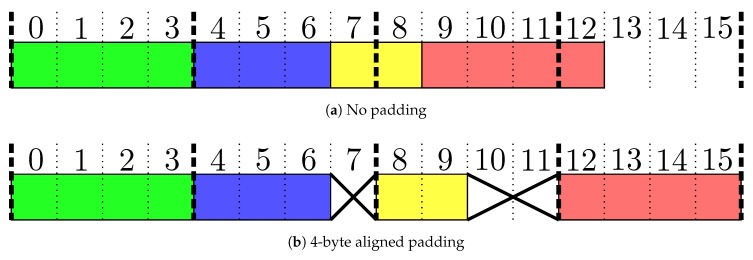
Four queued packages with and without padding. The crosses represent dont-care data, which may be zeroed out.

To enable efficient data transfer, processors can use DMA for data transfers. The DMA module is responsible for copying a set of data from one part of the processors memory to either another part of the memory, or to an external interface such as the USB interface. Without DMA the CPU needs to explicitly copy one word at a time from one memory address to another, which is inefficient both because the CPU could do something more useful, and because it might not be fast enough to keep up with higher transfer rates. A downside with some DMA implementations is that the starting address must be aligned in memory. Figure 6a shows a visualization of 16 bytes of memory with four queued messages, shown in four different colors. Now we imagine that these messages are transferred one by one using DMA with a 4-byte alignment requirement. First the green package is transmitted: the index is 0, the four bytes are written as expected, and the index is increased to 4. Then the blue package is transmitted: index is 4, three bytes are transmitted, and the index is increased to 7. Now, the yellow package is transmitted, but since the index is 7, it is no longer on the boundry and the DMA rounds the index down to the closest alignment and two bytes from there, resulting in that bytes 4 and 5, instead of 7 and 8 are transferred. Then the index is increased with 2 from 7 to 9. The next package (red) is 4 bytes long, but the index is still not aligned. Hence, the DMA copies 4 bytes from index 8 instead of from index 9, resulting in another erroneous transfer.

A proposed implementation that efficiently handles both messages of varying size and rate, and limitations in accessible memory addresses is given in Listing 1. The queueing implementation assumes the following interface with the processor’s USB-interface: a USB-transfer is initiated with a function named usb_write_dat that takes a pointer to the buffer to be written and the number of bytes to write from that buffer. Only one USB-write can be active at a time. When the transfer is finished, the USB-subsystem calls a function named usb_write_finished with a parameter containing the number of bytes that were written by the USB-interface. The number of bytes are less-than or equal to the requested number of bytes, but it is assumed that this number is always aligned to the specific interval specified by the DMA module of the processor. Furthermore, two functions for disabling and enabling interrupts on the processor, called usb_interrupt_disable and usb_interrupt_enable are assumed available. The memcpy_mod function copies data from a regular array to a ring buffer and is defined in Listing A.1 in Appendix A.

To address the problem due to DMA-alignment, code in Listing 1 automatically aligns each queued package to a 4-byte offset by padding the end of each package with up to 3 dummy-bytes, referred to as the extra variable in the code and the **PAD**-bytes in Figure 5. This allows any number of packages to be transmitted using DMA, allowing the system to fill the DMA-buffer if enough data is queued, without introducing issues with alignemnt, as every package is ensured to end at an aligned index. This approach is visualized in Figure 6b.

The write_scheduled variable does not need to be protected by the critical section, as it does not alter the behavior of the system, even though it is shared between the functions. If the variable is false when the function is called, no write events are in progress and thus the system cannot be interrupted by a finished write. The only exception to this is after a write has been scheduled at the very end of the function, after which the variable is no longer used. If the variable is true when entering the critical section, a write event should not be scheduled by the usb_queue_package function, and the write_scheduled remains unchanged.

The code provided in the listing is thread-safe under the following two assumptions: the usb_queue_package function is only called from the main loop, and the usb_write_finished is only called by the USB module when it has finished writing.


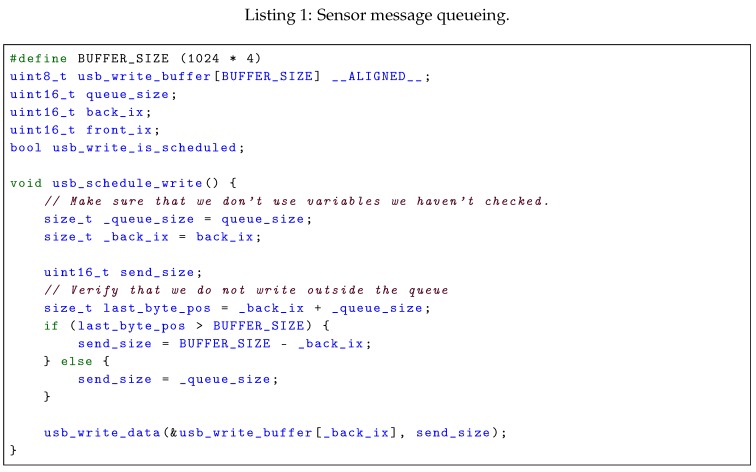



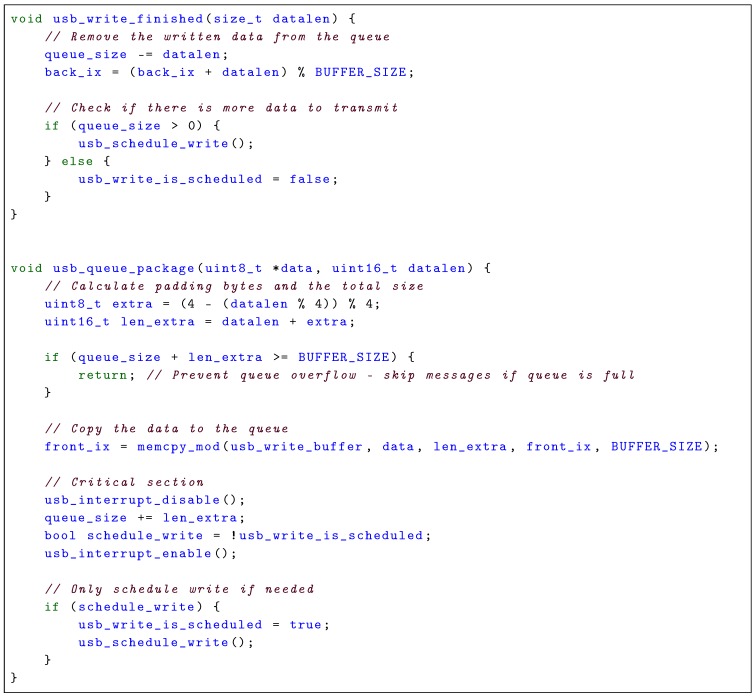


As the queue_size variable is changed both in the usb_queue_package and usb_write_finished functions, it needs to be protected in the critical section of usb_queue_package. A temporary variable, write_should_be_scheduled, is also created in this section. This variable indicates if the usb_queue_package should schedule the next write event, or if a write event is already in progress and the next write event should be scheduled in USB callback routine.

#### Microprocessor Considerations

When implementing this method on a microprocessor special considerations to the time spent in ISRs must be taken. Although the processor primarily runs in one main loop, this loop can be temporarily paused when handling ISRs. When an interrupt event occurs, the microprocessor sets a flag that the interrupt event has occurred and then launches an ISR and clears the flag. If multiple interrupt events happen simultaneously the associated flags are set and ISRs are executed in order of priority. If two interrupt events of the same type happen while the microprocessor is handling a higher-priority interrupt event, the flag for the lower-priority interrupt event can only be set once, and one of the events are dropped. To prevent loss of data or other events, the time spent in ISRs should be minimized.

This method minimizes the time spent while in the usb_write_finished-ISR, and the only time the data transfer could be further increased is when the end of the queue is reached, as only the data from the back_ix to the end of the queue is transferred. By choosing a sufficiently large QUEUE_SIZE, and thus reducing the frequency of the queue overflows, the loss of transfer capacity is negligible.

### 3.5. Sensor Configuration

As a developer of a sensor system is not necessarily an expert embedded system development, a hardware sensor timing system must be reconfigurable without impacting the overall system operation. To ensure that the system operation is intact, the developer needs to configure the hardware timing system without rewriting the firmware itself.

**Figure 7:**
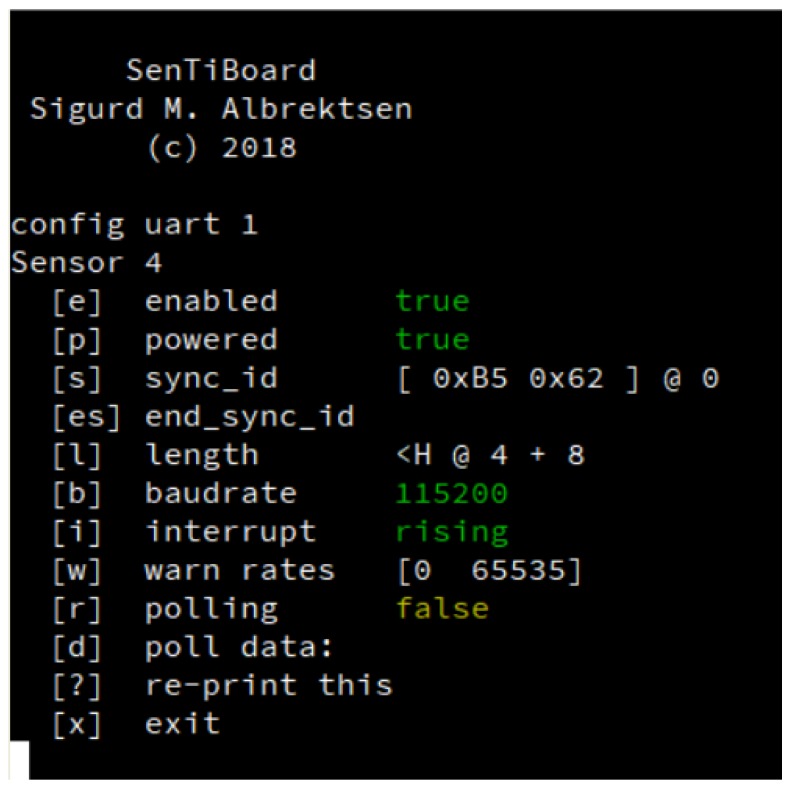
An example of a CLI for sensor port configuration.

To configure a hardware sensor timing system, we suggest providing two distinct methods: using a USB command-line interface (CLI), and using configuration files on an SD-card or other removeable storage. The main benefit of a USB command-line interface method is that it does not require any additional software except a standard VT102 terminal emulator such as Minicom [26] or PuTTY [27]—everything else is contained on the timing system itself. This method is ideal if changes must be made in the field or when configuring a new sensor for the first time, as the changes happen immediately. The configuration can then be written to the microcontroller’s non-volatile memory, so the configuration is kept even if the system is restarted. A screenshot of a command-line version of a sensor configuration module is shown in Figure 7.

**Figure 8:**
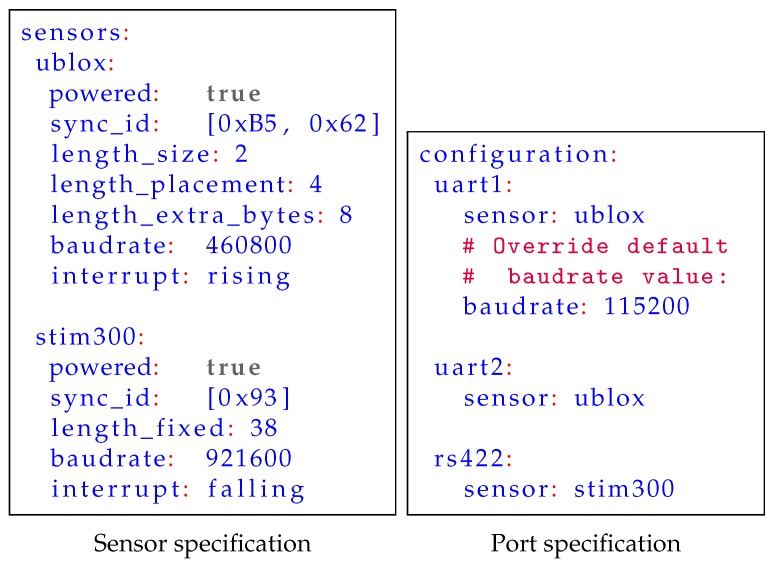
An example of text files for sensor port configuration, written in YAML.

The second method of configuring a hardware sensor timing system is to use configuration files placed on an SD-card or similar storage. This is an efficient method to use when identical sensors, or at least sensors with identical interfaces, are used in multiple sensor suites. To allow reuse of a configuration, the configuration for a specific sensor can be stored in a distinct file. This configuration can then be referenced to in the main configuration file, even several times if multiple identical sensors are connected simultaneously. An example of textual configuration files are shown in Figure 8. Reuse of sensor configuration files decreases system integration time, reduces the chance of errors as fewer files need to be changed.

## 4. SenTiBoard

**Figure 9:**
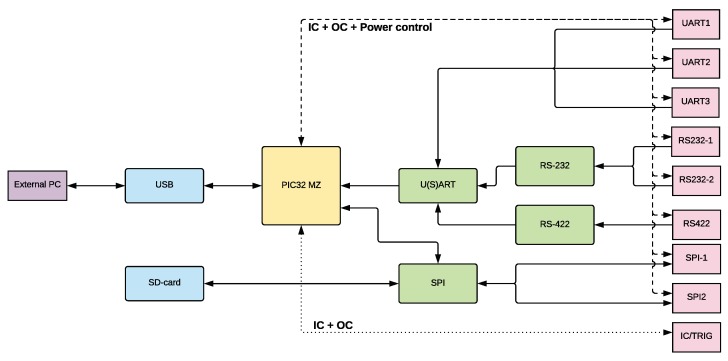
Overview of the hardware of the SenTiBoard. Each of the sensor ports (pink) have a IC-input pin, a OC-output pin and a power control pin in addition to pins for the associated protocol. The pink IC/Trig port only contains an IC-input pin and an OC-output pin.

To verify the proposed design, we have implemented the SenTiBoard (Sensor Timing Board), which is a reconfigurable sensor timing system that uses a PIC32MZ microprocessor running at 200 MHz, with a 32-bit timing counter running at 100 MHz to which the sensor readings are referenced. Communication with the SenTiBoard from an embedded or desktop computer is done through a double-endpointed High-Speed USB 2.0 interface; the first endpoint is used for configuration and debugging, and the second endpoint is used for sensor data transfer. If needed, for example if using an onboard computer without USB, sensor output can be redirected through one of the sensor ports. To record accurate timestamps from sensors, the SenTiBoard has a total of 9 IC-pins–allowing the system to simultaneously capture the TOV triggers from 9 sensors simultaneously. In addition, each sensor port can be triggered using the output compare (OC) functionality of the microcontroller. The physical dimensions of the board are 60 mm × 50 mm, and to simplify integration with the ODroid XU4, the removable adaptor legs shown in Figure 1a are attached to the board, with mounting holes that align with those of the ODroid. An overview of the SenTiBoard’s hardware is given in Figure 9.

By making the system reconfigurable, the firmware itself can be kept more stable, and creating specialized versions for every sensor combination can be avoided. This allows the firmware to be reused and maintained by experts that know the system well, while still being adaptable to the sensor-suites by non-experts, which again leads to fewer errors as shown by Mohagheghi et al. [28]: “Our results showed that reused components have lower defect-density than nonreused ones (almost 50% less)”.

For sensor communication, the SenTiBoard supports several protocols. By default, three ports with U(S)ART support, two ports with RS-232 support, one port with RS-422 support, and two SPI ports are implemented. Additionally, the three UART ports can be configured to use the I${}^{2}$C, also know as TWI, protocol, and other protocols can also be interfaced with the system. The SenTiBoard can also generate pulses that act as triggers for sensors. This is useful for example for triggering when a camera should record a photo, or to sample multiple IMUs simultaneously.

Power control is another feature of the SenTiBoard. Each sensor port has a controllable power output pin on either 3.3 V (for the SPI-ports), or 5 V (for the other ports), which are independently controlled by the microcontroller. This allows the SenTiBoard to turn on and off sensors as needed; for example, if a sensor is misbehaving and needs to be restarted, or in an emergency where the sensor needs to be powered off. There are also a set of error-LEDs, one per sensor port in addition to one for the whole system, that are used to indicate errors on the corresponding sensor and general errors.

The SenTiBoard has been previously used with a variety of sensor configurations in several experiments on unmanned aerial vehicles (UAVs), unmanned surface vehicles (USVs), and manned aircraft [29]. Note that earlier versions of the SenTiBoard were named SyncBoards.

### 4.1. SenTiUtils

To support system integration with the SenTiBoard and the SenTiStack (see Section 4.2), a utilities software package named SenTiUtils has been created. This package consists of documentation and software that interacts with the SenTiBoard, and software that converts the binary sensor data into higher level formats preferred by end-users, such as MATLAB’s .mat-format and NumPy for Python’s .npy files. The SenTiUtils package is split into five modules: Documentation, Testing, Logging, Parsing, Supervisor.

#### 4.1.1. Documentation

The documentation module contains details about the SenTiBoard hardware, the connectors used, how to configure the system, and the general structure of the intended usage of the SenTiBoard and the SenTiUtils package itself. This module also contains 3D-models of the board and connectors, sensor port specifications and mounting information. In addition, recommended configurations and tips for integrating commonly used sensors are provided.

#### 4.1.2. Testing

The testing module contains software for testing basic interaction between the SenTiBoard and the host computer. There are two scripts in this module; sentiboard_rate.py and senti_package_print.py. The sentiboard_rate.py script reads all sensor packages from the SenTiBoard and prints a list of the rate of each of the incoming packages every 0.5 s. This gives the user a quick overview to see if the packages are received as expected. The senti_package_print.py script can be used when integrating a new sensor with the SenTiBoard. This prints the bytes contained in each sensor message in a human readable text format which again can be compared and verified with the expected output from the sensor described in the manual or datasheet provided by the sensor’s manufacturer. Note that this module has nothing to do with testing the firmware of the SenTiBoard, but rather testing of the sensor configuration and communication.

#### 4.1.3. Logging

The logging module consists of a logging system which is recommended to use with the SenTiBoard. The logging system serves five main functions. Firstly, it creates a folder structure that ensures that logs are not overwritten when the system is restarted, by separating data in to different flights when it starts. Secondly, it separates messages from each sensor into distributed streams and distinct files. The number of sensor messages received by each sensor is printed by the logging system. This allows a user to supervise that the SenTiBoard and the logging system operates as intended by checking the log messages and the file system of each sensor, and allows the user to only parse data from the sensors needed for their application. Thirdly, the logging system creates a new logging-folder at specified intervals, set to 15 minutes by default. The intention behind this time-splitting is to limit the impact of a file becoming corrupted which might happen if the onboard computer is powered off abruptly. Fourthly, the logging system provides an interface that automatically starts both the logging system itself and other necessary software for the sensor system when the onboard computer boots. Examples of such software are imaging logging software and logging of sensors not connected to SenTiBoards. Finally, the logging system injects a timestamp from the onboard computer into the stream of sensor data received by the onboard computer from the system. This allows the sensor packages on the SenTiBoard to be synchronized with messages not connected to either the SenTiBoard or a GNSS-receiver, for example when using cameras without external triggering pins.

#### 4.1.4. Parsing

The parsing module contains code responsible for interpreting the binary stream or stored files from the sensors, wrapped by the SenTiBoard, to data processable by sensor fusion algorithms and other applications. There are two implementations of this system, one written in Python and one written in C++. The Python implementation is primarily used for converting to MATLAB and Python NumPy formats, while the C++ implementation is intended for use for relaying messages to other systems such as ROS [30], DUNE [31] and the SenTiBoard supervisor system. To be extendible to support a variety of sensors, the parsing module consists of two parts: a general package reader that extracts the timing and metadata from the SenTiBoard envelope, and an interface for the specialized parses which is specific for each sensor type. The sensor data is then transmitted in a *Message* structure, which is specific to each sensor. To streamline integration of sensors of the same type, generic message types such as *ImuMessage* or *GnssMessage* can be used to handle IMUs and GNSS receivers from different manufacturers without having to change the external code. These message types can be extended as needed.

#### 4.1.5. Supervisor

The supervisor module consists of a graphical visualization tool for showing real-time data as it is received from the SenTiBoard. This tool does not have any large framework dependencies, such as ROS, so it is valuable when using the SenTiBoard without such frameworks. The tool receives parsed sensor messages and visualizes the data in one of three main views. The first view shows a map with both the current and historical position data from the GNSS-receiver, the second view either shows a plot of the acceleration, gyroscope and magnetometer readings from the IMU(s) and magnetometer(s). The third view shows current camera images, and is only active if a camera is connected. If a sensor fusion algorithm, such as an extended Kalman filter (EKF) is used, the first and second view can show the estimated position, the attitude and the heading instead of the measurement data.

### 4.2. SenTiStack

When developing new sensor fusion algorithms, having access to a high-quality reference is of great value. By comparing the results to a reference, an indicator on the performance of the newly developed algorithm can be obtained. The SenTiStack is a high-accuracy navigation payload that is designed to work with the SenTiBoard, and it serves two functions: primarily it provides high quality sensors and a high-accuracy navigation solution, but it is also a fully working and maintained example on how to configure and use the SenTiBoard. In addition to providing a solution, additional sensors can be added at will, except for the ports the sensors for the SenTiStack occupy. Sensors that have integrated with the SenTiStack so far are autopilots, RGB, IR and hyperspectral cameras, air data/pressure sensors, phased array radio navigation equipment and specialized sensors created at our university, but other system can be integrated as well. A benefit with using the SenTiStack as a reference system when developing algorithms that in addition to provide a navigation solution, also provides the sensor data the estimates are based on, so that a new algorithm can be tested with identical sensor input.

**Figure 10:**
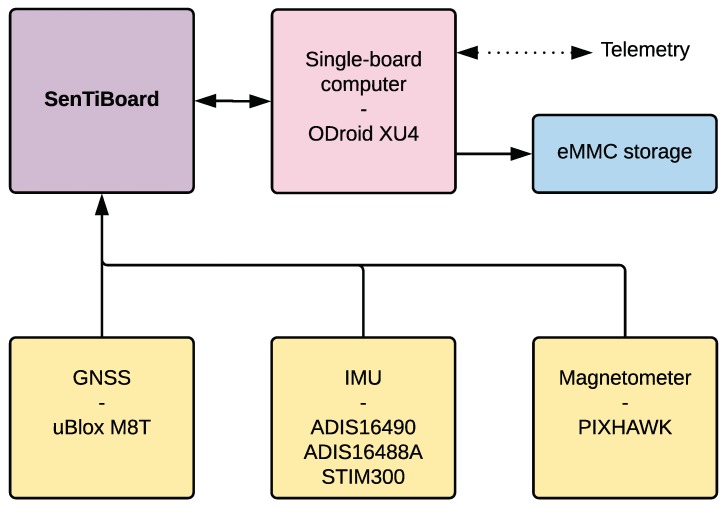
Overview of the components of the SenTiStack. In each of the sensor boxes (yellow), one or more of the options can be chosen, depending on the specific mission requirements. The telemetry link is optional, but can provide useful data about the system status, current sensor measurements, and provide data for RTK-GNSS calculations.

Although the intended use is for UAVs, the SenTiStack has been used on other platforms such as ground rovers, unmanned surface vehicles (USVs) and manned aircraft. The SenTiStack consists of a SenTiBoard, an ODroid XU4 single-board computer, one or more uBlox M8T GNSS receivers and an IMU. Several different IMUs have been used; the Sensonor STIM 300 [32], the Analog Devices ADIS16490 [33], the Analog Devices ADIS16488A [34], and a combination of multiple IMUs simultaneously. In addition, a magnetometer connected to the UAV’s autopilot has been used. An overview of the hardware of the SenTiStack is given in Figure 10.

As the SenTiStack is used with a specific set of sensors, additional support for this system can be included. This includes pre-made parsers and integration with middleware systems such as ROS and DUNE. Furthermore, we are currently implementing a state estimator, onboard the SenTiBoard, and this will primarily support the sensors on the SenTiStack. By recording multi-GNSS pseudorange, Doppler, carrier phase, phase lock and signal quality information from the onboard GNSS receiver, and recording correspo nding data at a local base station, a highly accurate RTK-solution can be calculated using the open source solution RTKLIB [35]. By using the RTK-GNSS solution to aid IMU and magnetometer measurements in a non-linear observer, or an EKF, position velocity attitude (PVA) estimates can be obtained [1]. Thus, the SenTiStack is developed to be a complete high-accuracy navigation and timing system with tactical grade sensors, a hardware sensor timing board, and sensor fusion algorithms.

### 4.3. Verification

The operation of the implemented hardware sensor timing system was verified in two ways. First, the queueing method’s maximal transfer rate was tested by transmitting dummy sensor messages with a data size and rate distribution typically seen in a sensor configuration. Then the clock accuracy was tested with a UAV experiment, where a GNSS receiver was used to provide a TOV and timing solution.

#### 4.3.1. Transfer Rate Verification

To verify that the SenTiBoard’s throughput using the proposed queueing strategy is sufficient, a  performance benchmark was performed. In this benchmark a dummy sensor suite is implemented in the firmware. This dummy-suite only sends mocked data packages, but the size and rate of the sensor messages are based on real sensors. The following imagined sensors with the specified messages are connected: a uBlox M8T GNSS receiver transmitting a navigation solution at 1 Hz and raw measurements at 10 Hz with sizes 100 bytes and 648 bytes respectively (based on UBX-NAV-PVT and UBX-RXM-RAWX with 20 satellite readings (pp. 307, 337 [36]); a Sensonor STIM300 [32] with rate, acceleration, inclination and temperature, transmitting 58 bytes at 2500 Hz; and two ADIS16490 transmitting 32 bytes (sync-id, temperature, delta-angles, delta-velocities and status) at 5000 Hz. The values 2500 Hz and 5000 Hz were chosen for convenience because they are the closest to a 5000 Hz counter already implemented on the SenTiBoard, and these values are sufficiently close to the real sensor values of 2000 Hz and 4250 Hz for testing purposes. In addition to the bytes from each sensor message, the 22–25 bytes from the SenTiBoard envelope are added to each package, depending on the number of *extra* bytes.

**Figure 11:**
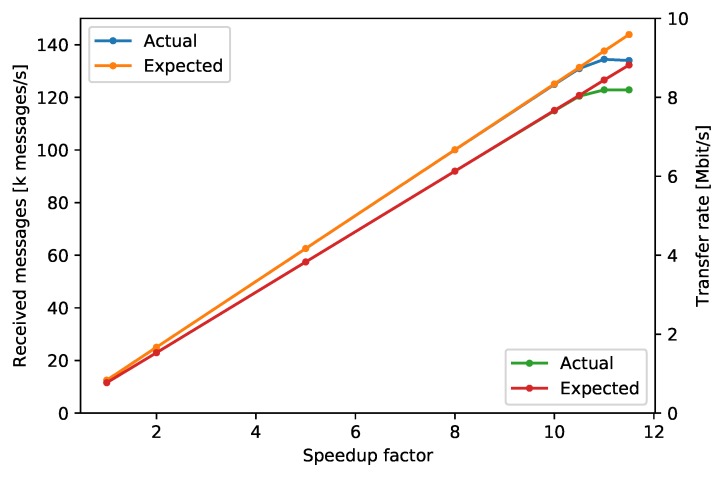
Visualization of Table 3.

With the test-configuration given above, the SenTiBoard could transfer all the packages as required. To test the maximal transfer speed of the SenTiBoard, we gradually increased the rate of the timer with until it reached a factor of 10, and the system still worked as expected, with very minor variances due to startup and shutdown timings. At this rate a total of 100,000 ADIS messages, 25,000 STIM messages and 110 uBlox messages are handled every second, and the total transfer speed is 7.66 MiB/s. When we further increased the speedup factor beyond 10.5, the system started to drop a significant number of packages, and we can see the data transfer does not increase further. See Table 3 and Figure 11 for details. The received messages are likely to be slightly different from the expected messages as some time is used to start and stop the process.

#### 4.3.2. Accuracy Analysis

**Figure 12:**
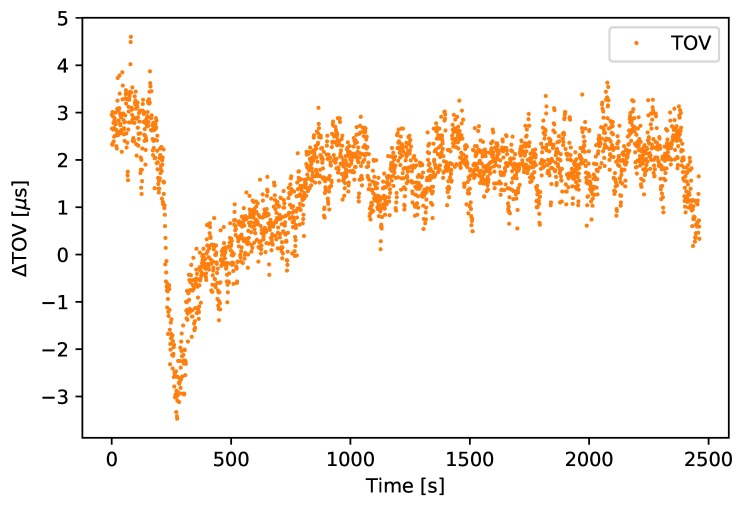
TOV accuracy, in microseconds.

To analyze the accuracy of the SenTiBoard’s timestamping an experiment was carried out, where a flight with the SenTiStack onboard a Skywalker X8 fixed-wing UAV, was performed. The uBlox M8T GNSS receiver was set to output a position velocity time (PVT) solution and corresponding TOV signal with a rate of 1 Hz, and reported an accuracy of 3.35 ns RMS during the flight. To calculate the relative accuracy, the difference between each TOV message was calculated. An RMS difference of 1.90 μs to the expected 1 s output from the GNSS receiver was found, and the result from the flight is shown in Figure 12. This inaccuracy is likely due to a drift in the oscillator and can be reduced, either by choosing a more stable primary oscillator, by attaching a stable secondary oscillator, or by increasing the output rate of the GNSS receivers’ TOV signal, assuming that the drift of the GNSS receiver is negligible. In addition, one may compensate for clock drift using a mathematical model for the drift due to variations of the temperature [37].

**Figure 13:**
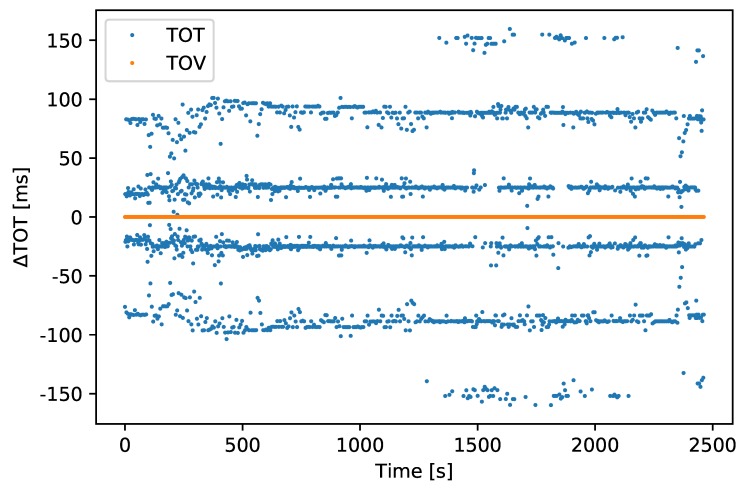
TOT accuracy vs. TOV accuracy, in milliseconds.

Without using hardware synchronization and recording the sensor’s TOV, the most common way of timestamping messages is to record the time when the first byte has been transferred (TOT). This method is inaccurate as it depends on several factors, such as internal processing time, the number of messages transferred before the desired message is transmitted, the size of these messages, and the transfer rate from the sensor. The RMS difference of the first received byte of the GNSS PVT message to the expected 1 s rate was 53.56 ms and the TOV and TOT values are visualized in Figure 13.

**Figure 14:**
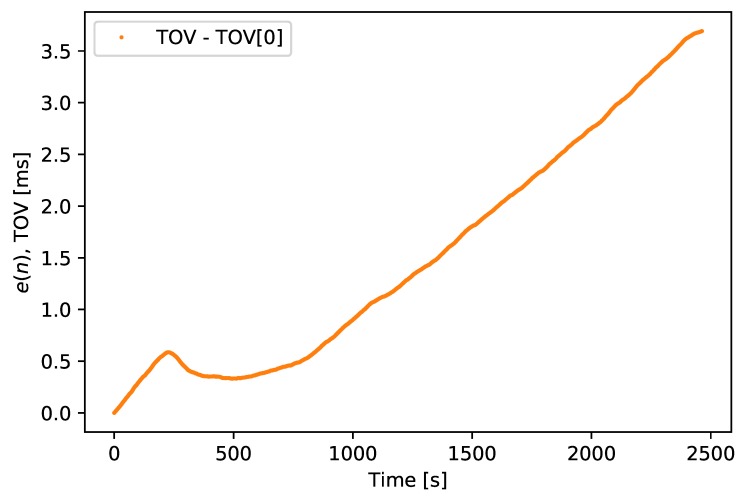
e(n) for TOV recordings, measured in milliseconds.

**Figure 15:**
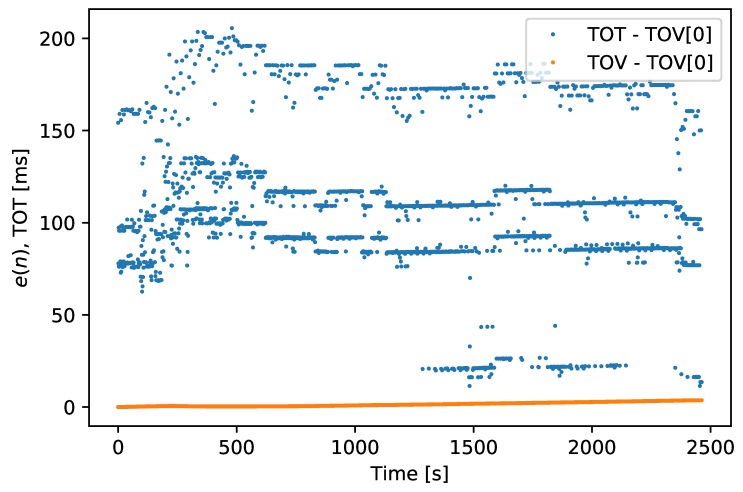
e(n) for TOT vs. TOV, measured in milliseconds.

To compare the accumulative effect of these inaccuracies over time, the following calculation was performed:

(1) e(n)=t(n)−(TOV(0)+n)

where t(n) is the TOV or TOT value *n* seconds into the dataset and TOV(0) is the first TOV recording. The e(n) of the TOV timestamp is shown in Figure 14, and the e(n) of the TOT timestamp is shown in Figure 15, with the e(n) of TOV for comparison. This error starts at the first measurement and then measure how much the proceeding measurements varies from the expected 1 s per measurement. As the measurement rate from the GNSS receiver is synchronized with a GNSS clock, it should be both precise and drift free. Hence, e(n) provides an estimate of the drift of the SenTiBoard’s clock over time.

#### 4.3.3. Camera Payload

To illustrate the use of the SenTiBoard in a complete remote sensing system, another experiment was carried out using the SenTiStack. This time with a SenTiBoard, an ODroid XU4 onboard computer, an ADIS 16490 IMU, a STIM 300 IMU, two uBlox M8T GNSS receivers and a FLIR Tau2 longwave infrared thermal camera. The main goal of this experiment was to track two surface vehicles, a manned boat and the Maritime Robotics Telemetron USV, using an infrared camera onboard a Cruiser-Mini fixed-wing UAV. The reason for having two GNSS antennas in this payload is to be able to estimate the heading of the UAV. To achieve a high quality RTK GNSS solution, an additional GNSS receiver, in addition to the two GNSS receivers onboard the UAV, was placed at the base station. This base station is located at a fixed point and used as a reference to compensate for atmospheric disturbances.

**Figure 16:**
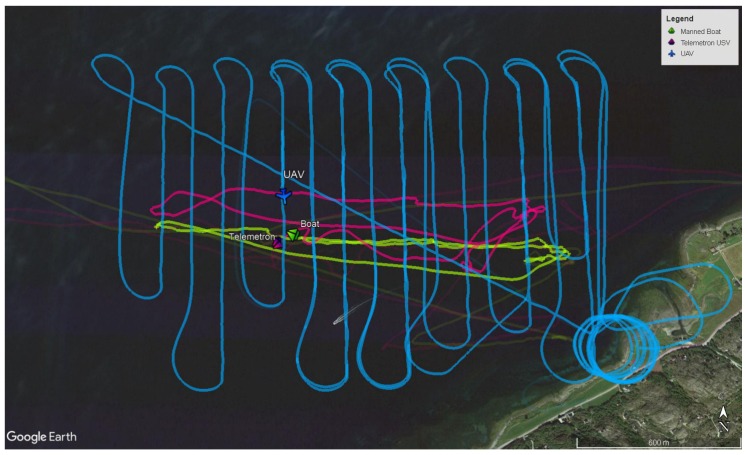
A partial GNSS track of the UAV’s and the two surface vehicles’ position.

**Figure 17:**
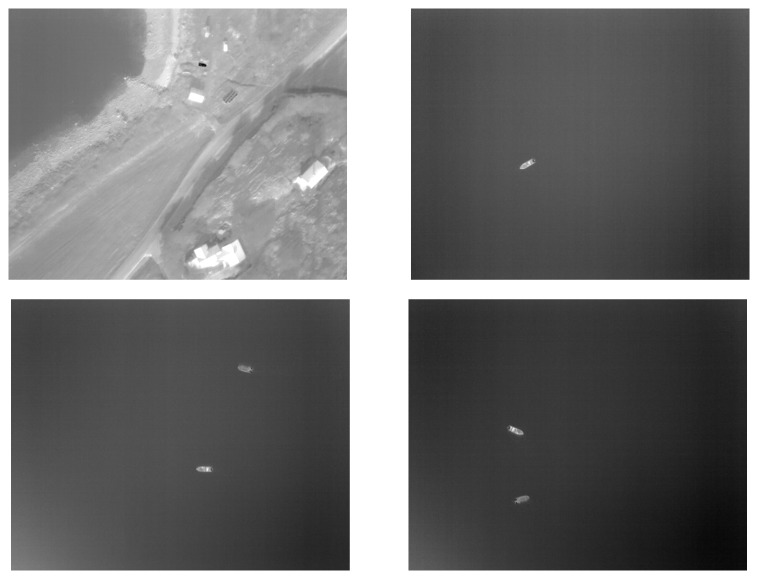
Infrared images captured during the mission.

To accurately synchronize the camera images with the UAV’s position, the SenTiBoard was used to trigger an image capture circuit connected to the camera, using the OC functionality of the SenTiBoard. As the OC is connected to the same hardware timer running the microcontroller as the IC, the accuracy of the trigger signal is comparable to the results found in Section 4.3.2. The capture circuit attaches a timestamp, in milliseconds, to each camera image, and the timestamp can be set to zero using an external input pulse. To synchronize this input pulse with a GNSS receiver, the SenTiBoard is configured to emit an output pulse which is synchronized with a TOV pulse from one of the attached GNSS receivers, although at a higher framerate. This pulse is sent to the input trigger of the timing cirucuit board, which then makes the associated timestamp from the capture board synchronized with GNSS-time. A partial track of the position of the USVs and the UAV are visualized in Figure 16 and examples of infrared images captured during the mission are shown in Figure 17.

An analysis of the results from this experiment is given in  [38] where the authors estimate an improvement in georeferencing errors from 10 m–15 m at an altitude of 100 m, to a mean of 9 m at an altitude of 350 m–400 m, when comparing to a solution with the same camera without GNSS synchronization. When accounting for the altitude difference, this corresponds to an accuracy improvement of a factor of 4, as improved accuracy is achieved at 4 times the altitude. This accuracy improvement is due to several factors, such as improved navigation and camera synchronization.

## 5. Conclusions

The high speed and fast dynamics of unmanned aerial vehichles make them valuable assets in many applications today, but these features also pose a challange when it comes to sensor accuracy. As we want UAVs to perform more complex tasks, such as performing hyperspectral imaging, high accuracy is needed—not only for the sensor values, but also in the time domain. The rapid movements a UAV is capable of make an attached sensor to also move quickly, and thus cause large inaccuracies if the associated timestamp is imprecise.

This paper describes required and desirable features of a reconfigurable hardware sensor timing system. An overall system design, a method for assigning timestamps to sensor messages using a data envelope, an approach for receiving sensor data and efficiently transmitting timestamped messages has been described. By making the solution reconfigurable, both the software and the hardware of the sensor timing system can be kept stable, which increases the reliability of the system, without compromising on sensor accuracy. Two methods are suggested for reconfiguration: one based on a command-line interface, and one based on files stored on a removable media such as an SD-card.

To verify the functionality of the suggested system a hardware implementation, the SenTiBoard, has been created. The system has a 32-bit, 100 MHz timer and uses interrupt capture to accurately record timestamps. It supports several commonly used protocols, power control of individual sensor ports, and triggering of external sensors such as cameras. In addition to the system itself, a support system that consist of both software for testing and verification, and hardware solutions for providing a rapid navigation solution for UAVs, consisting of a RTK-GNSS capable GNSS receiver, one or two IMUs, and a magnetometer has been created.

The implementation has been tested and a maximal transfer speed of more than 8 MiB/s was achieved, allowing the board to transfer more than 130,000 packages per second. A relative accuracy of 1.90 μs per second was measured, and an absolute accuracy of 2.75 ms after 2000 s when not compensating for the drift. An experiment with a hardware synchronized thermal camera was also performed and georeferencing errors were reduced with a factor of 4, compared to a software synchronization solution.

### Future Work

For future work, it would be interesting to investigate how to not only raw sensor measurements, but in addition create a configurable approach to parse and use sensor data in an onboard sensor fusion algorithm. This would allow the sensor timing board to provide attitude and position data instead of only positional data. It would also be valuable to have an embedded RTK-GNSS solution running, either on the microcontroller or by attaching an external co-processor, to improve the accuracy of the GNSS solution.

With the emerging industrial focus on precision agriculture, we believe that agricultural aerial and ground vehicles along with hyperspectral imaging can highly improve current methods based on regular RGB and multi-spectral imaging. An approach where a position and attitude solution is calculated using a double or triple RTK-GNSS solution, would be a valuable addition to the current state of the art, and should be researched further.

## Figures and Tables

**Table 1:** Comparison of hardware capabilities of embedded systems.

Name	Type	Clock Speed	Input Captures	U(S)ART/SPI	USB
Arduino MEGA	MDS	16 MHz	1	4/1	External only
Arduino Due	MDS	84 MHz	6	3/1	2.0 High-speed
Teensy	MDS	180 MHz	2	6/3	2.0 High-speed
Intel Edison	MDS	500 MHz	0	2/1	2.0 High-speed
Raspberry Pi 3 B+	Single-board	4 × 1.4 GHz	0	2/1	2.0 High-speed
ODroid XU4	Single-board	4 × 2 GHz	0	1/1	3.0 SuperSpeed
PIC32 MZ	Microcontroller	252 MHz	9	6/6	2.0 High-speed

**Table 2:** ADIS IMU product line gyro stability comparison [20].

Part Number	Release Date	Gyro Stability	Max Sample Rate
16485	Dec. 2012	6.25${}^{\circ }$/h	2460 Hz
16488A	May 2014	5.1${}^{\circ }$/h	2460 Hz
16490	Apr. 2017	1.8${}^{\circ }$/h	4250 Hz
16495	Nov. 2017	0.8${}^{\circ }$/h	4250 Hz

**Table 3:** Average transfer speed and number of messages received during a 10 second period. Each value is the average of 5 consecutive recordings.

Speed Factor	Received Messages	Expected Messages	Transfer Rate	Expected Rate
1	125,178	125,110	0.77 MiB/s	0.77 MiB/s
2	250,278	250,220	1.53 MiB/s	1.53 MiB/s
5	625,430	625,550	3.83 MiB/s	3.83 MiB/s
8	1,000,597	1,000,880	6.13 MiB/s	6.13 MiB/s
10	1,249,119	1,251,100	7.66 MiB/s	7.67 MiB/s
10.5	1,309,422	1,313,655	8.03 MiB/s	8.05 MiB/s
11	1,344,635	1,376,210	8.19 MiB/s	8.44 MiB/s
11.5	1,340,067	1,438,765	8.19 MiB/s	8.82 MiB/s

## Abbreviations

The following abbreviations are used in this manuscript:

CPU	central processing unit
DMA	direct memory access
EKF	extended Kalman filter
GNSS	global navigation satellite system
GPS	global positioning system
I${}^{2}$C	inter-integrated circuit
IC	input capture (also known as interrupt capture)
IMU	inertial measurement unit
IR	infrared radiation, used to specify cameras recording in the infrared spectrum
ISR	interrupt subroutine
OS	operating system
PVA	position velocity attitude
PVT	position velocity time
RGB	red green blue, used to specify cameras recording images in human visible colors
RS-232	recommended standard 232
RS-422	recommended standard 422
RTK	real-time kinematic
SPI	serial peripheral interface bus
TOA	is the time-of-arrival—the time when a full sensor message has been transferred to the receiver
TOT	is the time-of-transport—the time when the first byte of a data package is received
TOV	is the time-of-validity—the time when a sensor reading was validated; often referred to as PPS (Pulse-per-second) in timing applications
TWI	two-wire interface
U(S)ART	universal (synchronous and) asynchronous receiver-transmitter
UAV	unmanned aerial vehicle
USB	universal serial bus
USV	unmanned surface vehicle

## Appendix A. Ring Buffer Memcopy

Support methods for copying to and from ring buffers are described in Listing A.1. Note that the implementation on the SenTiBoard are optimized to avoid using the modulo operator which has a high computational cost.
